# Hyperimunoglobuline e syndrome: case report

**DOI:** 10.1186/1939-4551-8-S1-A53

**Published:** 2015-04-08

**Authors:** Lara Abdo, Fernanda Nodari, Tatiana Basso Biasi, Franciani De Oliveira Basso

**Affiliations:** 1 Universidade Federal De Santa Catarina, Brazil; 2 Prefeitura Municipal De São José, Brazil

## Background

Hyperimmunoglobulin E syndrome (HIES) is a rare desease caused by a primary complex immunodeficiency, with IgE serum levels over 2,000U/mL. The syndrome is characterized by the following triad: 1) recurrent staphylococcal abscesses, 2) recurrent airway infections; 3) elevated serum IgE concentration. Our pacient presented signs that are compatible with HIES in distinct moments of his life – microabscesses associated with eczema at adolescence, respiratory infections from childhood until present moment and seric IgE level of over 2,000 in recent dosage.

## Methods

This is a case report.

## Results

55 year old male complains of intense pruritus over entire body, with 100ºF fever and shiver episodes when pruritus was more intense, associated with 11kg wheight-loss during one year. During first year of life, he presented furunculosis; at adolescence, featured diffuse eczematous lesions, highly pruritic and associated with frequent impetiginisation. Pacient has asthma since childhood, with two previous hospitalizations due to pneumonia.

At dermatologic exam, pacient presented hyperkeratotic erythematous-desquamative plaques, with edema, erosions and desiccated serous crusts, as well as fissures and ulcers. Linear excoriation signs and xeroderma were present in all integument. IgE dosage evidenced levels over 2,000 U/mL, without especified exact value. The result of pathological exam presented spongiotic and psoriasiform superficial dermatitis with proeminent eosinophils.

Several therapeutic approaches have been tried in the past, all insuccessful. It was then prescripted Metotrexate with folic acid and prednisone – pacient responded with significant clinical improvement, especially decrease of pruritus. The dose of prednisone was progressively decreased until 40mg/day associated with Metotrexate 7.5mg 3 times/week and folic acid 5mg 4 times/week. Pacient persists with important decrease of lesions and consequent improvement of life quality.

## Conclusions

Cutaneous infections offen happen, and furunculosis and celulitis may be observed. “Cold abscesses”, which are neither erythematous nor painful, occur mainly in pacients that are not submited to prophylactic antibiotic therapy; they are pathognomonic of HIES, though not indispensable for diagnosis. Recurrent respiratory infections cause pulmonary sequelae that lead to chronic respiratory failure, the main cause of death in patients with this desease.

HIES is a condition whose diagnosis is hard, mostly due to its rarity. Frequently, pacients pass by several medical institutions and go through partially or completely insuccessful therapeutic approaches, what delays even more the correct diagnostic conclusion and the beginning of effective treatment. Nevertheless, after the introduction of appropriate immunossupressive therapy, pacients tend to stabilization of clinical condition and important improvement of life quality.

## Consent

Written informed consent was obtained from the patient for publication of this abstract and any accompanying images. A copy of the written consent is available for review by the Editor of this journal.

